# Automatic Identification of Glomerular in Whole-Slide Images Using a Modified UNet Model

**DOI:** 10.3390/diagnostics13193152

**Published:** 2023-10-09

**Authors:** Gurjinder Kaur, Meenu Garg, Sheifali Gupta, Sapna Juneja, Junaid Rashid, Deepali Gupta, Asadullah Shah, Asadullah Shaikh

**Affiliations:** 1 Chitkara University Institute of Engineering and Technology, Chitkara University, Rajpura 140401, Punjab, India; gurjinder.kaur@chitkara.edu.in (G.K.); meenu.garg@chitkara.edu.in (M.G.); sheifali.gupta@chitkara.edu.in (S.G.); deepali.gupta@chitkara.edu.in (D.G.); 2 Kulliyyah of Information and Communication Technology, International Islamic University Malaysia, Kuala Lumpur 53100, Malaysia; asadullah@iium.edu.my; 3 Department of Data Science, Sejong University, Seoul 05006, Republic of Korea; junaid.rashid@sejong.ac.kr; 4 Department of Information Systems, College of Computer Science and Information Systems, Najran University, Najran 55461, Saudi Arabia; shaikhasad@hotmail.com

**Keywords:** deep learning, detection, glomerular, kidney tissue, UNet, whole-slide images

## Abstract

Glomeruli are interconnected capillaries in the renal cortex that are responsible for blood filtration. Damage to these glomeruli often signifies the presence of kidney disorders like glomerulonephritis and glomerulosclerosis, which can ultimately lead to chronic kidney disease and kidney failure. The timely detection of such conditions is essential for effective treatment. This paper proposes a modified UNet model to accurately detect glomeruli in whole-slide images of kidney tissue. The UNet model was modified by changing the number of filters and feature map dimensions from the first to the last layer to enhance the model’s capacity for feature extraction. Moreover, the depth of the UNet model was also improved by adding one more convolution block to both the encoder and decoder sections. The dataset used in the study comprised 20 large whole-side images. Due to their large size, the images were cropped into 512 × 512-pixel patches, resulting in a dataset comprising 50,486 images. The proposed model performed well, with 95.7% accuracy, 97.2% precision, 96.4% recall, and 96.7% F1-score. These results demonstrate the proposed model’s superior performance compared to the original UNet model, the UNet model with EfficientNetb3, and the current state-of-the-art. Based on these experimental findings, it has been determined that the proposed model accurately identifies glomeruli in extracted kidney patches.

## 1. Introduction

As many health-associated problems are increasing each day all around the world, kidney diseases are also growing rapidly. Mainly, these diseases are caused by other health problems such as diabetes, cholesterol, and glomeruli diseases [1]. The histological study of glomerular diseases requires a detailed analysis of digital kidney slides, including the detection and evaluation of each glomerulus. The identification of the glomeruli is important for kidney disease diagnosis at an early stage because the glomeruli purify the whole blood of the body at least 40 times a day [2]. During filtering, it removes the waste products from the blood and keeps the required substances in it. Damage to these glomeruli tissues is a common symptom of kidney problems, which can eventually lead to kidney failure [3]. As these glomerular diseases are caused at the cellular level, biopsies are performed at the same level to identify their abnormalities [4]. In kidney biopsies, a small sample of kidney tissue is extracted using a needle and examined under a microscope to identify the glomeruli [5]. However, the process of visually analyzing kidney biopsy slides using traditional methods, such as a microscope, is a difficult task [6].

Nowadays, the introduction of automated slide processing has led to significant advancements, improving the effectiveness of conventional procedures and facilitating the achievement of more objective and standardized diagnoses [7]. This automation phenomenon proves highly advantageous within healthcare environments, specifically in institutions such as hospitals, regions, or countries with a shortage of nephropathology professionals [8].

With machine learning and deep learning, significant improvements have been achieved in the digital pathology domain, which focuses on diagnosing and quantifying diseases by analyzing medical images acquired from scanned pathological tissue samples [9,10]. This study uses deep learning techniques to identify glomeruli in human kidney tissue slides.

The proposed work discusses a UNet-based framework to automatically identify glomeruli in digital Periodic Acid Schiff (PAS)-stained whole-slide images (WSIs) in kidney tissue biopsies. The proposed model enabled us to locate the glomeruli in images by attaining better outcomes than the existing work regarding accuracy, precision, recall, and F1-score. In this article, the major contributions of this research work are as follows:

1. The UNet model is modified by changing the number of filters and feature map dimensions from the first layer to the last layer for deep feature extraction. Moreover, the depth of the UNet model is also enhanced by adding one more convolution block to the encoder as well as the decoder section. To accurately identify the glomerular position in the kidney images, two convolution layers, one batch normalization layer, and one max pooling layer were added to the encoder, and one convolutional layer, one upsampling layer, and one concatenate layer were added to the decoder.
2. To achieve better results, the proposed model was tuned with different hyperparameters like optimizers, epochs, and batch sizes.
3. The performance of the proposed model was evaluated in terms of accuracy, precision, recall, and F1-score. Moreover, its performance is compared with different state-of-the-art models.

The remaining paper is structured as follows: Section 2 describes the related work, and Section 3 represents the materials and methodology used in the study. Section 4 describes the results analysis, and Section 5 concludes the paper.

## 2. Related Work

Identifying glomeruli in digital images of human kidney biopsies enables us to determine whether the glomeruli in kidneys are healthy or diseased. If the glomeruli are diseased, they can cause kidney failure, and if there is an insufficient number of healthy glomerulus in the kidney, this can indicate that the kidney is unsuitable for transplantation. Nowadays, the techniques for classifying and detecting glomeruli have become more important for research and kidney disease diagnosis. Several authors have worked on different methods to detect glomeruli in kidney tissue images. Some research papers work is presented here: Cascarano et al. [11] proposed a computer-aided design system that used a feature-based approach to classify the glomeruli into sclerotic and non-sclerotic forms. After training the model, the glomeruli detection was performed and achieved a precision of 98% and a recall of 93%. Kannan et al. [12] developed a CNN model to differentiate between NPS and GS images. The proposed CNN model was trained with 1362 cropped input images and labels, which led to an accuracy of 92.67% and a Kappa score of 0.86%. This study concluded that deep learning was the best method for analyzing digital human kidney images with complex histological structures.

Zeng C. et al. [13] proposed a deep-learning method for identifying glomeruli and glomerular lesions in PAS-stained whole-slide images. For all the different glomeruli variants, the model achieved a precision of 93.1% and an average recall of 94.9%. Chandan et al. [14] presented a CNN segmentation model to locate glomeruli in human kidney images using pattern recognition. This study compared the faster region-based CNN and mask-based CNN’s ability to detect glomeruli in dataset images. After training, the faster region network had 57.6% accuracy and 65.5% recall. By contrast, the mask region had 58.8% precision and 65.5% recall.

Ye Gu et al. [15] proposed a multi-stream glomeruli segmentation framework to quantify and classify kidney tissue features. To accomplish this task, they used different combinations of models. In one model, they combined ResNet with FCN and DeepLabv3 to improve data encoding. In the other model for the feature extraction task, the UNet model was modified using the efficient net as the backbone of the model. The Bayesian voting method did the best out of all the methods, with an F-score of 91.5%. Xuevai et al. [16] proposed the Mask RNN model by modifying deconvolution layers to segment the glomeruli in a pixel-level glomerular microscopic image dataset. After training the modified Mask RNN model, the results in terms of precision increased by 88.3%.

Altini et al. [17] proposed a framework to automatically detect and classify glomeruli in human kidney histological sections. The authors segmented the glomeruli in 26 images using the SegNet and DeepLabv3+ techniques and achieved an F1 score of 85.9% and 92.4%, respectively. Gallego et al. [18] proposed a CNN model for detecting glomeruli and a CNN with an AlexNet model to classify the glomeruli in 10,600 human kidney patches. The precision, recall, and F1-score were measured by evaluating the model on test images at 88.1%, 100%, and 93.7%, respectively.

Gadermayr et al. [19] worked on segmenting glomeruli regions using two distinct CNN cascade techniques and obtained a 90% dice coefficient value. After that, the results were also compared to fully convolutional networks, and it was concluded that the CNN cascade techniques were best suited for the segmentation of the glomeruli. Kato et al. [20] demonstrated the use of supervised classification models based on a linear Support Vector Machine (SVM) for segmenting glomeruli in rat whole-slide images of a kidney. To detect glomeruli, they used segmental and rectangular Histograms of Oriented Gradient (HOG) descriptors, and it was concluded that the Segmental HOG outperformed rectangular HOG in real-world testing regarding detection performance.

Temerinac-Ott et al. [21] presented the CNN-based technique to detect and recognize glomerular structures in WSls of histopathological slides stained with different reagents. The performance of CNNs on different stains was assessed. It provides a unique strategy for improving glomeruli detection on a single stain by considering the classification results from consecutive sections of the same tissue stained with different dyes. Comparing the model’s performance on four distinct stains, they determined that this integrative technique could increase the detection rate of a single stain by up to 30%. Ginley et al. [22] proposed a computer-aided diagnosis system to categorize kidney biopsies of diabetic patients using a mix of machine learning and standard image processing methods and achieved Cohen’s kappa 55%. Saikia et al. [23] proposed MLP (Multi-Layer Perceptron)-based architectures to segment glomeruli in PAS-stained WSIs and diagnose kidney diseases effectively. The proposed method employs MLP-UNet to segment glomeruli instead of conventional convolutions.

Shubham et al. [24] proposed a deep-learning technique to identify the glomeruli in digital kidney tissue sections. This study used the UNet segmentation model and EfficientNetB4 as its backbone. With the help of this combination, the authors measured the value of the dice coefficient at approximately 91%. Davis et al. [25] proposed a UNet architecture with nine layers of convolutional neural networks to segment the sclerotic and non-sclerotic glomeruli in the frozen section of human kidney donor biopsies. After training the model, they achieved F1, recall, and precision scores for non-sclerotic and sclerotic glomeruli at 93%, 96%, 90%, 87%, 93%, and 81%, respectively. Jiang et al. [26] developed cascade mask region-based convolutional neural network architecture to classify and divide glomeruli into three groups: normal glomeruli, glomeruli with lesions, and globally sclerotic glomeruli. The F1 scores measured for total glomeruli were 91.4%.

## 3. Material and Methods

The proposed model exploits the modified U-Net architecture to identify glomeruli in human kidney images. This model was assessed using HuBMAP hacking the kidney dataset, which comprised 20 whole-slide microscopy kidney images.

### 3.1. Datasets Description

**Figure 1:**
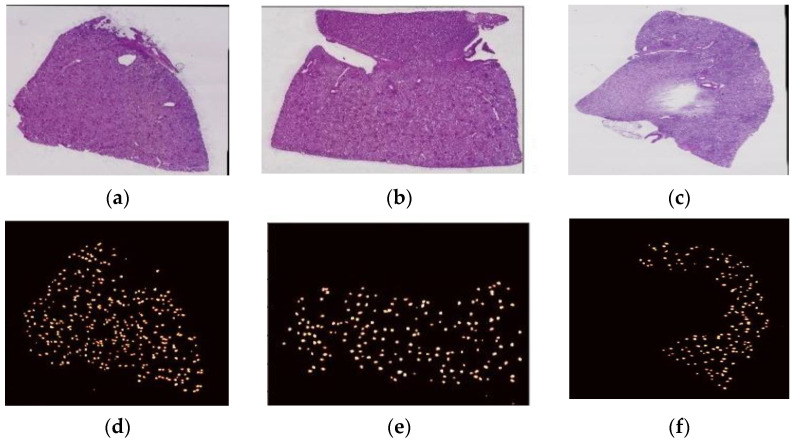
(**a**–**c**) Sample original whole-slide images, (**d**–**f**) Ground truth mask of the respective whole-slide images.

The dataset utilized in this study was obtained from Kaggle, from the HubMap hacking the kidney section. The dataset comprising 20 WSIs of kidney tissue was stained using the PAS reagent [27]. The images were produced at high resolution using a bright field scanner set at 20× magnification from the slides of kidney biopsies. As a sample, three WSIs from the dataset are shown in Figure 1, where Figure 1a–c illustrates the original WSIs of the dataset and Figure 1d–f displays the corresponding ground truth masks for the WSIs.

The dataset also includes the Run-Length-Encoded (RLE) file, which contains RLE codes for all segmented glomeruli [28]. These RLE codes were encoded to create a ground truth mask of segmented glomeruli in whole-slide images of kidney samples; these masks were further used to evaluate the model’s performance.

### 3.2. Extraction of Tiles from Whole-Slide Images

**Figure 2:**
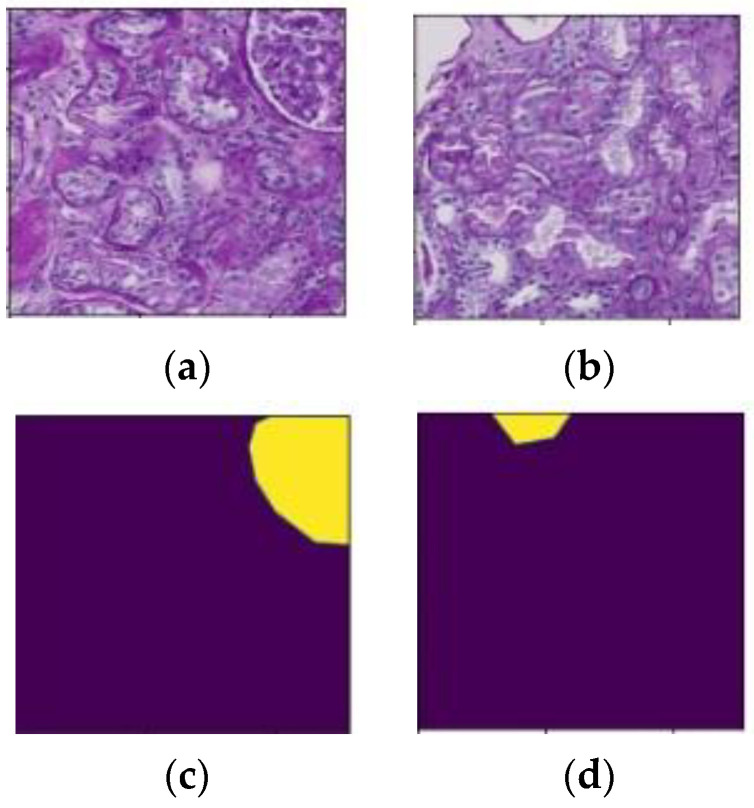
(**a**,**b**) Extracted tiles with a size of 512 × 512 pixels, and (**c**,**d**) Ground truth masks of the respective tiles.

Due to the large size of each WSI, it is impossible to use them directly; therefore, all WSIs and their respective mask sizes must be reduced [29]. All WSIs with an original size between 13,013 × 18,484 pixels and 49,548 × 38,160 pixels were cropped into 512 × 512-pixel tiles for this purpose. After cropping, the dataset consisted of 50,486 tiles. The entire collection of cropped tiles was divided into 26,874 training tiles and 23,612 testing tiles to train and test the model, respectively. Figure 2a,b show the 512 × 512 pixel extracted tiles, and Figure 2c,d show their ground truth masks.

### 3.3. Data Augmentation of Glomeruli Tiles

**Figure 3:**
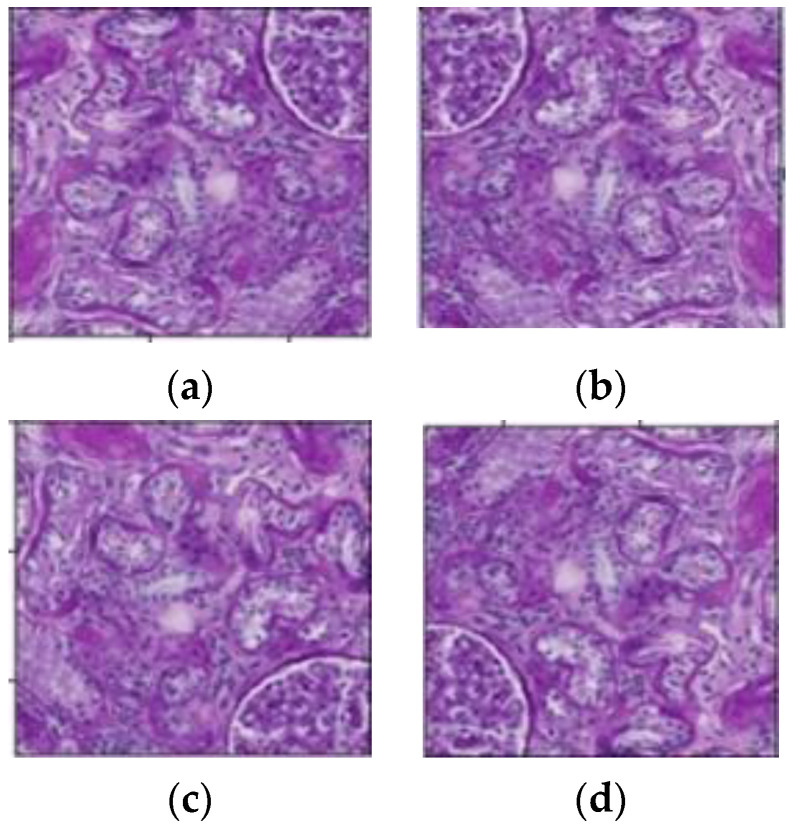
Tiles after the data augmentation technique (**a**) Original tile; (**b**) Horizontal-flipped tile; (**c**) Vertical-shifted tile (**d**) Horizontal-shifted tile.

The extracted 50,486 tiles included 13,441 glomerulus tiles that contained at least one glomerulus, while the remaining 37,045 tiles did not contain any glomeruli. The imbalance in the distribution of tiles (images) could result in the model being overfitted. To maintain a balance between the number of glomerulus and non-glomerulus images, the data augmentation method was used. The glomerulus images increased through the vertical shift, horizontal shift, and horizontal flip techniques. After augmentation, the dataset contained a total of 40,323 glomerulus images. Figure 3 depicts the various data augmentation techniques utilized in this study.

### 3.4. Proposed Modified UNet Model Implementation

**Figure 4:**
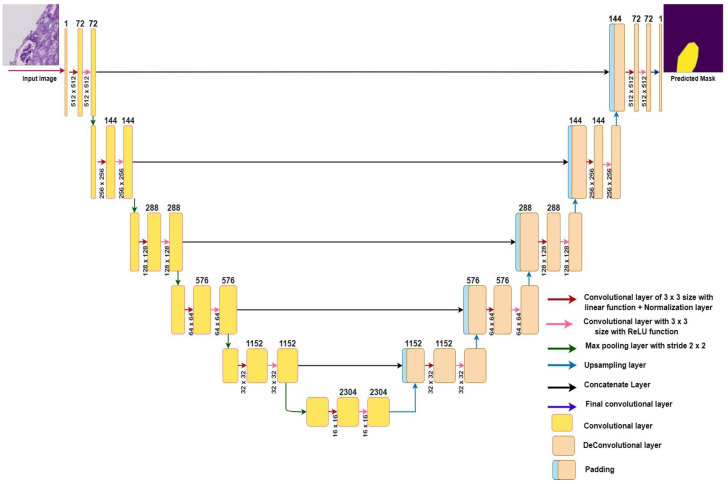
The architecture of the proposed modified UNet model.

This paper proposes a modified UNet-based framework to identify the area of the glomeruli in kidney tissues. Figure 4 depicts a modified version of the UNet model, wherein several key architectural aspects have been altered. These modifications encompass adjustments to the number of filters, feature map dimensions, and the inclusion of additional layers compared to the original UNet model.

A 3 × 3 convolutional layer with a linear function, a normalization layer, a 3 × 3 convolutional layer with a ReLU function, and a 2 × 2 max pooling layer were added to the encoder (contraction path) to pull out more features and patterns from the input image. A 3 × 3 convolutional layer, an upsampling layer, and a concatenate layer were added to a decoder (expansive path) to recover the spatial information from the input image. The addition of layers to the UNet architecture also increased the training time and the chance of overfitting. Usually, this occurs when there are few training images in the dataset. However, we already had a large dataset of images, and we also applied the data augmentation technique to increase the number of images.

Table 1 provides a analysis of the layers used in both the original UNet model and proposed modified UNet model, along with descriptions outlining the roles of these layers.

Figure 4 represents the different layers with different-colored arrows in the architecture of the modified UNet model. In the contraction path of the model, there were five blocks of layers; each block contained one convolutional layer with a normalization layer and another simple convolutional layer with the max pooling layer. In the initial block, the number of filters increased from 1 to 72. The green arrow indicates the max pooling layer, which reduced the feature map dimensions from 512 × 512 to 256 × 256 by decreasing the number of pixels in the output of the convolutional layer and increasing the number of filters from 72 to 144. To accomplish downsampling, this procedure was repeated four times. In the second, third, fourth, and fifth convolution operation, the feature map was reduced from 256 × 256 to 128 × 128; from 128 × 128 to 64 × 64; from 64 × 64 to 32 × 32; and from 32 × 32 to 16 × 16, respectively. At the end of the contraction path, the image was 16 × 16 and contained 2304 filters. As the image resolution became very low after the contraction path, it had to be converted back to a high resolution before the output, as the input and output should have the exact resolution [30]. To accomplish this, the model’s expansive path was utilized, which consisted of five convolution blocks. In each block, the upsampling layer, followed by the convolutional layer, was used to increase the image size and decrease the number of feature maps. By contrast, the concatenate layers were combined with the contraction path layers to obtain the image’s previous information.

In the first convolutional operation, beginning at the bottom, the upsampling layer halved the number of filters and doubled the image size from 16 × 16 to 32 × 32. The concatenate layer combined this enlarged image with the image of the contraction path to obtain previous feature information; this helps the model make more accurate predictions to localize glomeruli in the kidney patches [31]. The feature map was increased to 64 × 64 in the second block, 128 × 128 in the third, and 256 × 256 in the fourth block. Finally, the feature map size changed to 512 × 512 in the topmost block. After resizing the image, a convolutional layer was applied, and a glomeruli mask based on the input was generated at the output.

**Figure 5:**
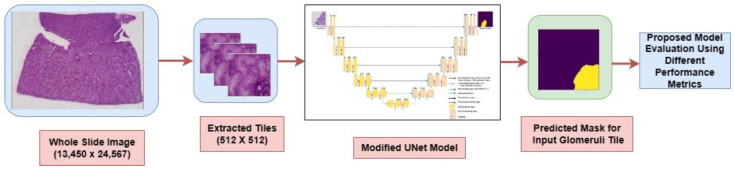
Overview of the proposed method to detect glomeruli in kidney patches.

Figure 5 illustrates the implementation methodology of the proposed modified UNet model. Initially, the original WSIs were cropped into tiles with dimensions of 512 × 512 pixels. These extracted tiles were used as the input for the modified UNet model that, in turn, generated a mask which predicted the location of glomeruli within the input image. The model’s performance was evaluated by comparing this predicted mask to the ground truth mask.

## 4. Result Analysis

This section presents a comprehensive analysis of the results obtained from three different models: the original UNet model, the UNet model with EfficientNetb3 as an encoder, and the proposed modified UNet model. The analysis focuses on comparing the various aspects of these models.

The performance evaluation of the models involved the computation of various performance metrics followed by a comparative analysis of the outcomes across different experiments. These metrics include classifications such as True Positives and True Negatives, as well as misclassifications like False Negatives and False Positives, all of which are derived from the confusion matrix. To measure the model’s accuracy, we assessed it by calculating the ratio of the total number of actual events to the sum of all pixel classifications, including both correct classifications and misclassifications [32]. Precision, on the other hand, was determined by quantifying the number of positive class predictions. Recall measured the ability of the model to correctly identify positive cases among all actual positive events, while the F1-score was computed using both recall and precision [33].

Initially, only the original UNet model’s performance was assessed in terms of validation loss and validation accuracy. Further, the original UNet model was modified using its encoder as a transfer learning model. For this, four transfer learning models were used, i.e., ResNet101, EffcientNetb3, VGG19, and DenseNet. The performance of the UNet model with these four transfer learning models as the encoder was assessed, and from their results, it was determined that EfficientNetb3 performed better than the other three transfer learning models. A detailed description of all the analysis is discussed below:

### 4.1. Analysis Using Different Optimizers

In the process of model training, optimizers play a crucial role in reducing the loss function and enhancing overall model performance. To validate the model’s performance with a batch size of eight and to determine the most effective optimizer, four different optimizers were employed: Adam [34], SGD [35], Adadelta [36], and RMSprop [37]. These optimizers were used to calculate validation loss and validation accuracy.

As shown in Table 2, a thorough comparison of different optimizers was performed for the original UNet model, the UNet model with EfficientNetb3, and the proposed modified UNet model. This analysis aimed to evaluate each model’s performance with different optimizers. With an epoch of 10 and batch size of eight, the Adam optimizer emerged as the standout performer, showcasing the lowest validation loss value and the highest validation accuracy when compared to the other optimizers. This superiority of the Adam optimizer was consistent across all three models, highlighting its effectiveness at optimizing model performance. Moreover, from Table 2, it can also be observed that, with the Adam optimizer, the proposed modified UNet model achieved the highest accuracy at 88.77 and lowest loss at 23.12 in comparison to other models.

### 4.2. Analysis Using Different Batch Sizes with Adam Optimizer

After selecting the Adam optimizer, the performance of the model was evaluated using different batch sizes. As shown in Table 3, batch sizes of 8, 16, and 24 were used, and evaluations were performed at epoch 10 to calculate validation loss and validation accuracy. This analysis was required to determine how different batch sizes affected the performance of the model in terms of these key metrics.

Table 3 shows that a batch size of eight provided low validation loss and high validation accuracy for all three models compared to other batch sizes of 16 and 24. Moreover, the proposed modified UNet model outperformed in terms of validation accuracy at 87.77 and validation loss at 23.12 compared to other models with a batch size of eight.

**Figure 6:**
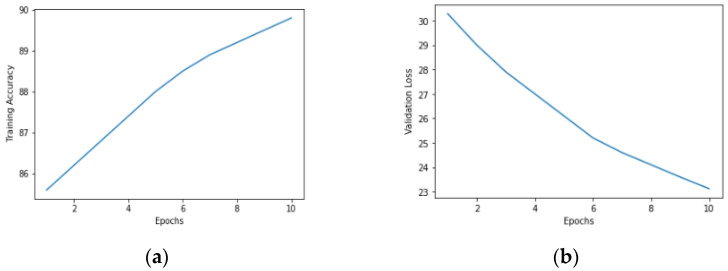
Plots of proposed modified UNet model with Adam optimizer, batch size eight and 10 epochs: (**a**) Validation accuracy (**b**) Validation loss.

The graphical representation of the validation loss and validation accuracy for the proposed modified UNet model using a batch size of eight and 10 epochs is illustrated in Figure 6a,b. Figure 6a demonstrates a positive correlation between the number of epochs and the validation accuracy. Figure 6b illustrates a decreasing trend in the loss value as the number of epochs increased.

### 4.3. Analysis Using Different Epochs with Adam Optimizer and Batch Size 8

After selecting the Adam optimizer and a batch size of eight, we evaluated the performance of all three models at epochs 10, 20, 30, 40, and 50. During these evaluations, we calculated validation loss and accuracy to observe the performance of the model during training. It can be observed from Table 4 that the validation loss had the lowest value at epochs 50, and the validation accuracy attained its highest value at the same epochs in all three models. However, at this time as well, the proposed model attained the best results in terms of validation accuracy at 92.6 and validation loss at 16.41 in comparison to other models.

### 4.4. Analysis of Proposed Modified UNet Model with Adam Optimizer, Batch Size 8 and Epochs 50

In the last section, the proposed modified UNet model demonstrated superior performance compared to other models when evaluated in terms of validation accuracy and loss [38,39]. This achievement was accomplished using the Adam optimizer, with a batch size of eight and 50 epochs. Further, in this section, the proposed model was analyzed visually and in terms of confusion matrix parameters like accuracy, precision, recall, and the F1-score to comprehensively evaluate the proposed model’s performance at this specific batch size and epoch count.

#### 4.4.1. Visual Analysis Based on Predicted Masks

**Figure 7:**
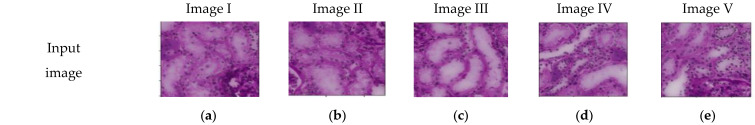

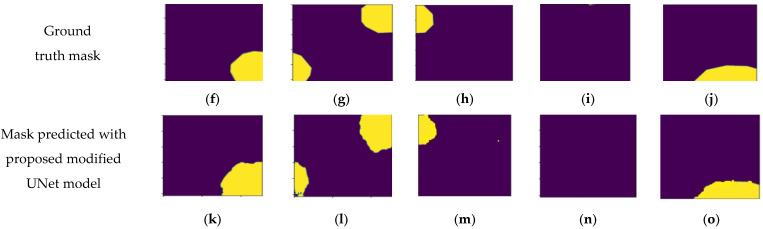
Images with Adam optimizer, a batch size of eight and 50 epochs: (**a**–**e**) Input images; (**f**–**j**) Ground truth masks of the respective input images; (**k**–**o**) Masks predicted by the proposed modified UNet model.

To evaluate the visual performance of the proposed model, a comparison was made between the ground truth masks and masks generated by the proposed model. Figure 7 provides a visual analysis using a selection of five sample images. In this analysis, the input images shown in Figure 7a–e and corresponding to these input images, Figure 7f–j, display the ground truth masks. Additionally, Figure 7k–o depict the masks predicted using the modified UNet model. This visual examination clearly represents how well the proposed model aligned with the ground truth masks for these specific sample images.

#### 4.4.2. Analysis Based on Confusion Matrices

**Figure 8:**
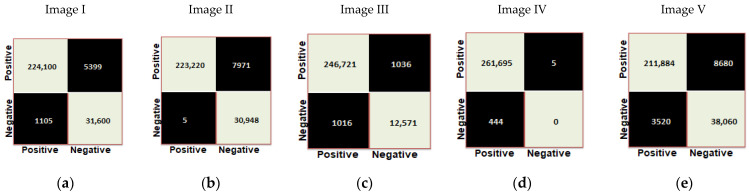
(**a**–**e**) Confusion matrices obtained for five sample images with Adam optimizer, a batch size of eight and epochs 50 using proposed modified UNet model.

Confusion matrices were generated for the proposed modified UNet model based on five sample images labeled as Image I, Image II, Image III, Image IV, and Image V. Figure 8 provides representations of the confusion matrices for the proposed model, while Table 5 presents an analysis of performance metrics for the proposed model for five sample images. With an average performance score of 95.7% for accuracy, 97.2% for precision, 96.4% for recall, and 96.7% for the F1-score on test images, it indicates that this model was effectively trained and evaluated and could be reliably employed for the accurate identification of glomeruli in human kidney images.

### 4.5. Comparison with State of the Art

Table 6 gives a summary of the studies identifying glomeruli based on species, types of staining, the number of WSIs, the number of cropped images, the technique used, and performance parameters. Moreover, in Table 6, different parameters computed during the identification of glomeruli, such as accuracy, precision, recall, and F1-score, were also compared with the state-of-the-art. The primary focus of these studies has been the detection or segmentation of specific regions or structures within glomeruli images. From the comparison table, it can be observed that the proposed modified UNet model, which has a large dataset of 50,486 images with a 512 × 512 pixel size, yields better results compared to the previous state-of-the-art methods, as shown in Table 6. After the completion of the proposed model training process, the accuracy, precision, recall, and F1-score of the model based on test images were 95.7%, 97.2%, 96.4%, and 96.7%, respectively. These results show that the proposed model can accurately identify glomeruli in kidney images.

## 5. Conclusions

The outcomes demonstrate that the proposed modified UNet model can automatically find glomeruli in the WSIs of human kidney tissue sections. The results were evaluated on a large dataset of 50,486 kidney images, and it was observed that the proposed modified UNet model produced higher performance measurement values when compared with state-of-the-art approaches. As the proposed modified UNet model focuses on glomeruli detection, it remains challenging to integrate it into a clinical workflow for automated analysis. Creating a seamless pipeline incorporating the glomeruli detection model and providing clinically relevant data could be a significant future objective. Also, instead of only histopathological images, other imaging techniques such as ultrasound or MRI can be used for additional information.

## Figures and Tables

**Table 1:** Different layers used in UNet model and proposed modified UNet model.

Name of Layer	Number of Layers in Original UNet Model	Number of Layers in Proposed Modified UNet Model	Role of the Layers
Convolutional	12	15	It enhances the model’s capacity to effectively capture complex features, which further helps the model to obtain detailed features for glomerular position identification.
Max pooling	4	5	It is used in the UNet architecture’s encoder to reduce the spatial dimensions of feature maps, which is useful for capturing larger-scale features in images.
Upsampling	4	5	In the decoder portion of the UNet architecture, the upsampling layer is used to increase the spatial dimensions of the feature maps. It helps in the recovery of spatial information lost during the downsampling operations of the encoder.
Normalization	4	5	The normalization layer is applied to each layer’s feature maps to stabilize and accelerate training by normalizing the activations in a small batch.
Concatenate	4	5	The inclusion of additional concatenate layers creates more opportunities for the decoder to integrate features from various scales or levels of abstraction. This can potentially enhance the fusion of low-level and high-level features, ultimately leading to improved accuracy in segmentation.

**Table 2:** Comparison of different optimizers for original UNet model, UNet model with EfficientNetb3, and the proposed modified UNet model using a batch size of eight and 10 epochs.

Optimizer	Validation Loss			Validation Accuracy		
	Original UNet Model	UNet Model with EfficientNetb3	Proposed Modified UNet Model	Original UNet Model	UNet Model with EfficientNetb3	Proposed Modified UNet Model
Adadelta	70.34	69.32	67.61	84.25	86.35	86.42
RMSprop	52.65	50.56	48.25	85.04	86.78	86.83
SGD	69.23	68.21	66.04	78.47	80.72	82.76
**Adam**	**29.67**	**27.45**	**23.12**	**85.28**	**87.98**	**88.77**

**Table 3:** Comparison of different batch sizes for original UNet model, UNet model with EfficientNetb3, and the proposed modified UNet model using Adam optimizer and 10 epochs.

Batch Size	Validation Loss			Validation Accuracy		
	Original UNet Model	UNet Model with EfficientNetb3	Proposed Modified UNet Model	Original UNet Model	UNet Model with EfficientNetb3	Proposed Modified UNet Model
8	**25.78**	**24.9**	**23.12**	**82.67**	**84.98**	**87.77**
16	27.7	26.90	24.05	82.34	83.39	84.02
24	26.56	25.90	23.02	80.32	82.79	84.79

**Table 4:** Comparison of different epochs for original UNet model, UNet model with EfficientNetb3, and the proposed modified UNet model using Adam optimizer and batch size of eight.

Epochs	Validation Loss			Validation Accuracy		
	Original UNet Model	UNet Model with EfficientNetb3	Proposed Modified UNet Model	Original UNet Model	UNet Model with EfficientNetb3	Proposed Modified UNet Model
1	34.45	33.28	30.28	80.67	82.02	84.19
10	25.78	24.9	23.12	82.67	84.98	87.77
20	22.56	21.95	20.06	83.2	86.03	89.9
30	20.78	19.34	18.33	84.67	88.90	91.3
40	18.86	17.04	17.15	86.17	90.19	92.1
**50**	**16.53**	**17.1**	**16.41**	**91.88**	**87.46**	**92.6**

**Table 5:** Performance metrics of proposed modified UNet model for five sample images.

Metrics	Accuracy (%)	Precision (%)	Recall (%)	F1-Score (%)
Image I	97.5	97.6	99.5	98.5
Image II	96.9	96.5	99.9	98.2
Image III	98.9	95.8	99.6	99.6
Image IV	99.8	99.9	99.8	99.9
Image V	95.3	96	98.4	97.2

**Table 6:** Comparison of the proposed modified UNet model with the state-of-the-art.

Ref.	Species	Staining	Number of WSIs	Number of Cropped Images with Size (pixels)	Technique Used	Performance Parameters			
						Accuracy (%)	Precision (%)	Recall (%)	F1-Score (%)
Cascarano et al. [11]	Human	PAS	26	2772 (656 × 656)	CAD	95	98.4	93.1	95.6
Ye Gu et al. [15]	**Human**	PAS	---	---	FCN+ ResNet/DeepLab v3	---	---	---	91.5
Altini et al. [17]	Human	PAS	26	2772 (656 × 656)	SegNet,	---	83.4	88.6	83.8
					DeepLab v3+	---	93.5	91.3	89.7
Kato et al. [16]	Rat	Desmin	20	200 × 200	R-HOG + SVM	---	77.7	91.1	85.9
					S-HOG + SVM	---	87.4	89.7	92.4
Davis et al. [25]	**Human**	PAS	258	24,133 (256 × 256)	UNet with 9 layers of CNN	---	90	96	93
Jiang et al. [26]	**Human**	PAS	---	1123	Mask region based CNN	---	---	---	91.4
Kawazoe et al. [40]	Human	PAS	200	4029 (1100 × 1100)	Faster R-CNN	---	93.1	91.9	92.5
		PAM	200	4029 (1100 × 1100)		---	93.9	91.8	92.8
		MT	200	4029 (1100 × 1100)		---	91.5	87.8	89.6
		Azan	200	4029 (1100 × 1100)		---	90.4	84.9	87.6
Simon et al. [41]	Human	PAS	25	1649 (576 × 576)	MrcLBP + SVM	---	91.7	76.1	83.2
Barros et al. [42]	Human	PAS/H&E	---	811	LoG + KNN	88.3	92.3	88	90.08
Lo et al. [43]	Human	PAS/H&E	40	3473	Faster-RNN	---	86.5	91.5	88.9
**Proposed model**	**Human**	**PAS**	**20**	**50,486** **(512 × 512)**	**Modified UNet** **model**	**95.7**	**97.2**	**96.4**	**96.7**

## Data Availability

Data will be made available on request from the corresponding author.
